# Contrast‐Enhanced Ultrasound‐Based Radiomics for the Prediction of Axillary Lymph Nodes Status in Breast Cancer

**DOI:** 10.1002/cnr2.70011

**Published:** 2024-10-18

**Authors:** Haimei Lun, Mohan Huang, Yihong Zhao, Jingyu Huang, Lingling Li, HoiYing Cheng, Yiki Leung, HongWai So, YuenChun Wong, ChakKwan Cheung, ChiWang So, Lawrence Wing Chi Chan, Qiao Hu

**Affiliations:** ^1^ Department of Ultrasound People's Hospital of Guangxi Zhuang Autonomous Region & Guangxi Academy of Medical Sciences Nanning Guangxi China; ^2^ Department of Health Technology and Informatics Hong Kong Polytechnic University Hong Kong China; ^3^ Department of Ultrasound, Guangxi Hospital Division of The First Affiliated Hospital Sun Yat‐sen University Nanning Guangxi China

**Keywords:** axillary lymph nodes status, breast cancer, contrast‐enhanced ultrasound, Radiomics

## Abstract

**Background:**

Breast cancer is the leading cause of cancer‐related deaths in the female population. Axillary lymph nodes (ALN) are a group of the most common metastatic sites of breast cancer. Timely assessment of ALN status is of paramount clinical importance for medical decision making.

**Aims:**

To utilize contrast‐enhanced ultrasound (CEUS)‐based radiomics models for noninvasive pretreatment prediction of ALN status.

**Methods and Results:**

Clinical data and pretreatment CEUS images of primary breast tumors were retrospectively studied to build radiomics signatures for pretreatment prediction of nodal status between May 2015 and July 2021. The cases were divided into the training cohorts and test cohorts in a 9:1 ratio. The mRMR approach and stepwise forward logistic regression technique were used for feature selection, followed by the multivariate logistic regression technique for building radiomics signatures in the training cohort. The confusion matrix and receiver operating characteristic (ROC) analysis were used for accessing the prediction efficacy of the radiomics models. The radiomics models, which consist of six features, achieved predictive accuracy with the area under the ROC curve (AUC) of 0.713 in the test set for predicting lymph node metastasis.

**Conclusion:**

The CEUS‐based radiomics is promising to be developed as a reliable noninvasive tool for predicting ALN status.

## 1 Introduction

Breast cancer (BC) was the most commonly diagnosed cancer and the leading cause of cancer‐related death among females worldwide in 2020 [1]. Axillary lymph nodes (ALN) are a group of the most common metastatic sites of BC [2]. The detection of ALN status remains an integral part of BC management, influencing the stage, treatment plan, and overall prognosis [3]. To date, ALN dissection (ALND) is the golden standard for determining the presence of lymphatic spread and removing ALN that possibly contain cancerous cells [4]. However, it gives rise to nonnegligible morbidities, such as potential morbidity of uncomfortable postoperative drains and seroma, neuropathy, limited arm abduction, lymphedema, and increased risk of cellulitis. Meanwhile, ALND might be overtreatment for patients with a low disease burden in the sentinel nodes [5]. In comparison to ALND, sentinel lymph node (SLN) biopsy entails lower rates of lymphedema and other postoperative morbidities. However, according to three prospective multi‐institutional clinical trials, SENTINA, SN FNAC, and the ACOSOG Z1071, the overall false‐negative rates of SLN were 13%–14%, exceeded the prespecified threshold of 10% [6, 7, 8]. Therefore, it is imperative to explore an noninvasive, accurate, and feasible preoperative method for predicting metastatic status of ALN, aiding in clinical decisions for BC. To date, the most appropriate imaging detection method for predicting metastatic status of ALN has not been established.

Radiomics is an image analysis technique that extracts quantitative features from medical images and can capture tumor characteristics at the cellular and genetic levels [9, 10, 11]. Thus, it is assumed that the high‐dimensional radiomics features extracted from medical images could capture invisible tumor characteristics, reflect underlying cellular biology, and provide prognostic and predictive information [12, 13]. In recent years, the feasibility of radiomics in the preoperative prediction of lymph node malignancy in some BC to reduce the subjectiveness and augment the predictive ability of medical images has been determined using ultrasound images [14, 15, 16]. However, analyzing contrast‐enhanced ultrasound (CEUS) images by radiomics has not yet to be reported. To address this, we aimed to develop and validate a CEUS‐based radiomics signature for evaluating ALN status in BC.

## 2 Materials and Methods

**FIGURE 1:**
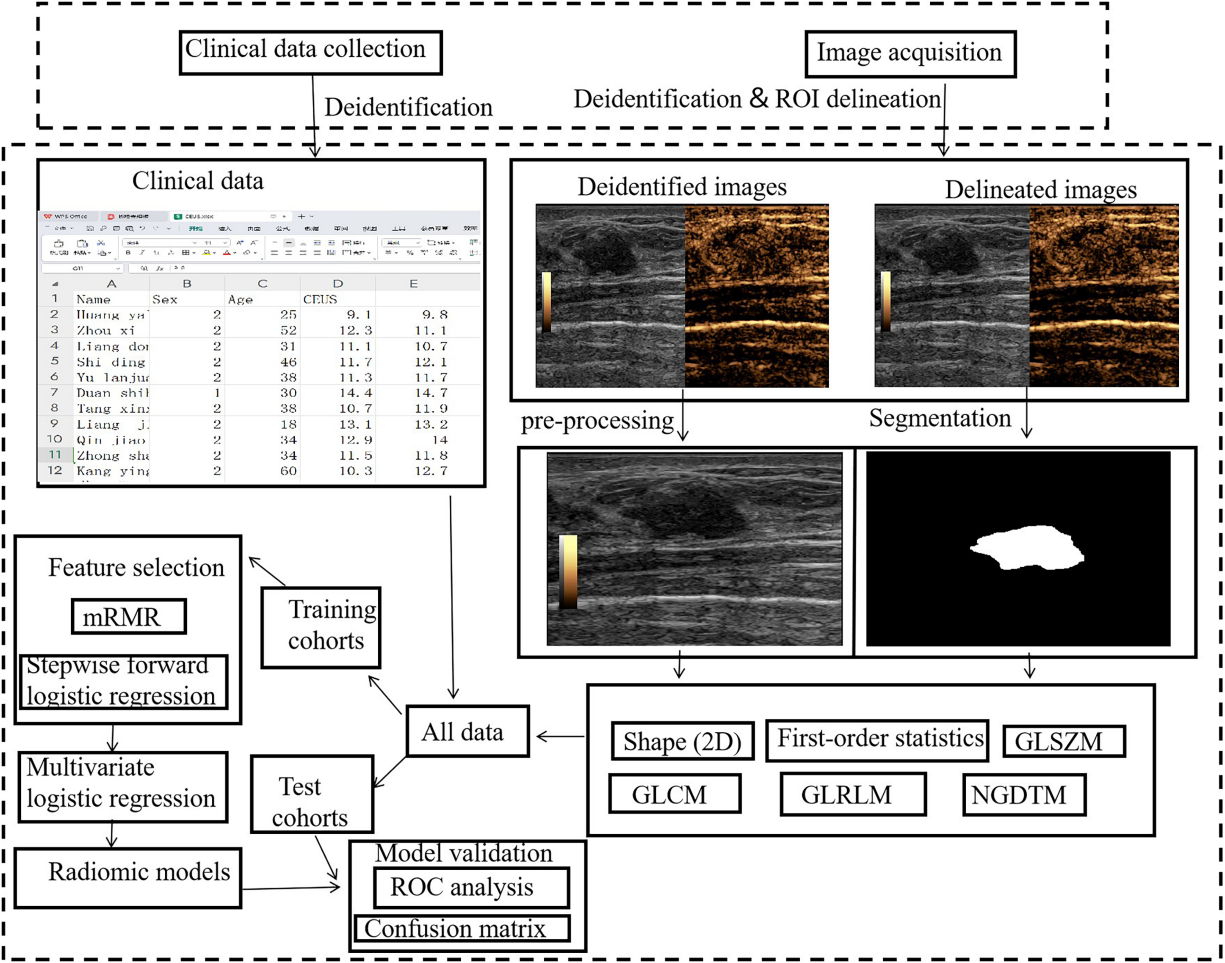
The study workflow.

This retrospective single‐center observational study was approved by the Ethics Committee of People's Hospital of Guangxi Zhuang Autonomous Region, China (No. KY‐LW‐2020‐24), which waived the requirement for informed consent. This study was divided into six parts, including data acquisition, image segmentation, feature extraction, feature selection, model construction, and model validation (Figure 1).

### 2.1 Study Population

From May 2015 to July 2021, a total of 293 patients with BC were enrolled in the study based on the following eligibility criteria: (1) female patients; (2) preoperative CEUS images with clear target lesions; (3) ALN metastatic involvement was pathologically confirmed by ALND in surgery; and (4) cases with complete clinicopathological information. The exclusion criteria: (1) male patients; (2) incomplete clinical data; and (3) blurred ultrasound images. All patients were diagnosed with ultrasound examination before any anticancer therapy and underwent surgical resection of tumor and ALND. Overall, 243 cases were assessed for model building in lymph node metastasis. Ten patients per feature were suggested to be reasonable for a radiomics model; therefore, the sample size in this study is reasonable [17].

Clinical data of the patients were also collected from the hospital's database. In particular, the ALN status of the patients was confirmed by the pathologists based on histopathological examination of lymph nodes retrieved during ALND and the patients were classified into node‐positive and node‐negative.

### 2.2 CEUS Examination and Segmentation

CEUS images of the primary tumors were acquired through different types of scanners using linear transducers (Table S1), with all parameters (such as depth, focus, and gain) set by the staff to achieve optimal image quality. CEUS images were collected in the format of the digital image with original quality.

**FIGURE 2:**
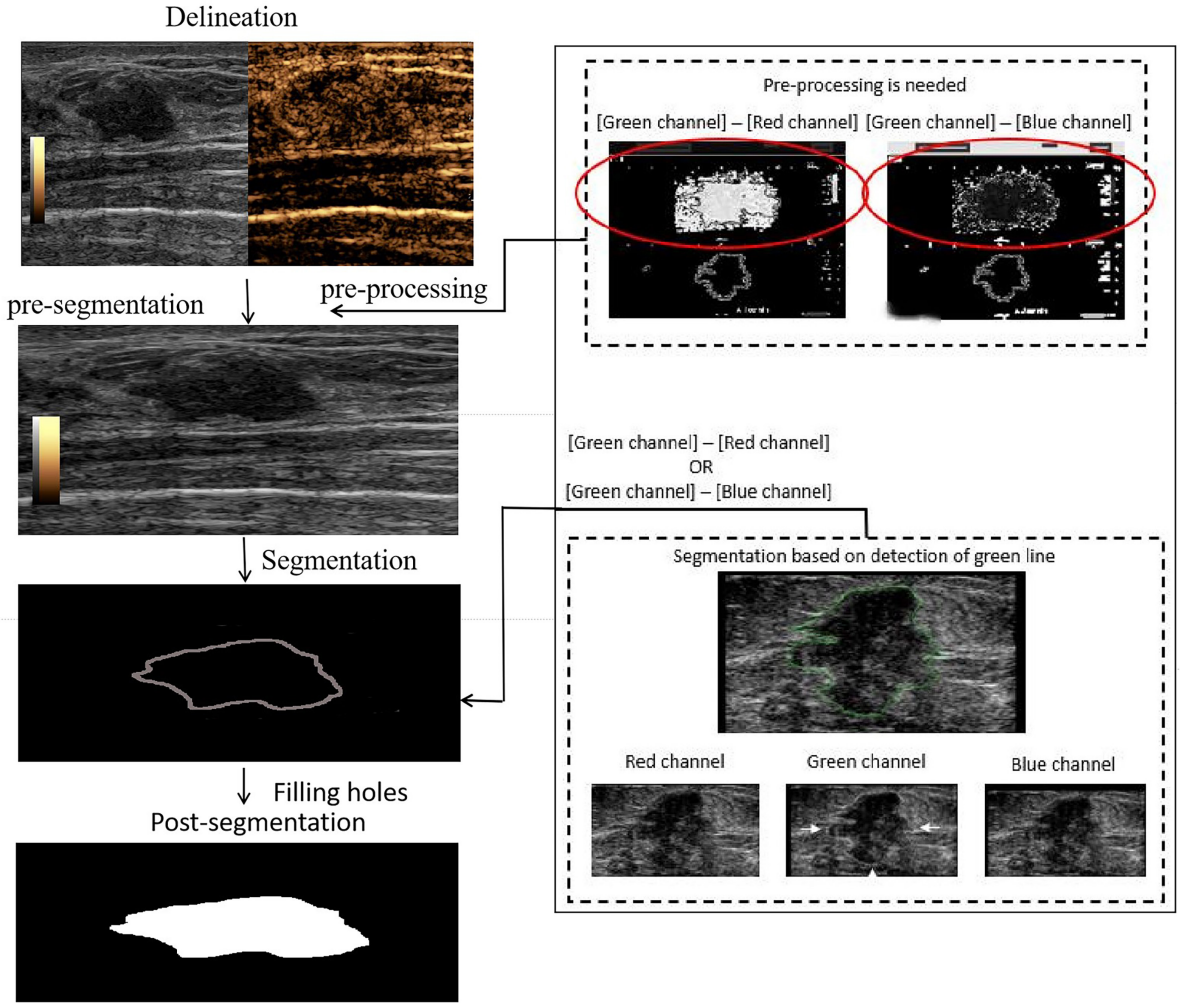
Flowchart of the CEUS segmentation. CEUS, contrast‐enhanced ultrasound.

The region of interest (ROI) was manually drawn with colored lines along the margin of the primary tumors by a junior sonologist with 3‐year clinical experience and reviewed by a senior sonologist with 16‐year clinical experience. The ROI delineations were repeated by the junior sonologist and the senior sonologist in 25 randomly selected cases to analyze the intraobserver and interobserver variations in manually delineated ROI and the impacts on the radiomics features. The spatial overlap between two segmentations was evaluated by the Dice similarity coefficient (DSC) [18, 19]. The DSC for all images is greater than 0.7, showing a good similarity of the delineated images from two segmentations. Intraclass correlation coefficients (ICCs) were calculated based on the feature extraction results to evaluate the impact of delineation variability on the repeatability of the features included in the radiomics signatures. Features with ICCs > 0.75 were considered robust [19, 20]. The ROI was segmented based on the detection of colored lines using Python (Figure 2).

### 2.3 Radiomics Feature Extraction and Selection

After obtaining the mask images and original images, features were extracted including gray level co‐occurrence matrix, gray level run length matrix, gray level size zone matrix, neighboring gray‐tone difference matrix, and gray level dependence matrix. The implementation details of the radiomics features are provided in the PyRadiomics documentation [17]. Finally, 474 radiomics features, including 9 shape‐based features, 18 first‐order statistics features, 75 second‐order statistics features, and 372 wavelet features were extracted from the sonologist‐delineated lesions for each case.

Randomization was performed to divide the cases into the training datasets and test datasets in a ratio approximating 9:1, which allows most images to be used for training the logistic regression models. This probably enhances the performance of the models at the expense of increasing the variance of the results [21]. Then, feature selection and model construction were performed on the training set, while the test set was used for model validation.

In this study, a two‐stage feature selection algorithm that combines a model‐free filter method and a classifier‐dependent wrapper method was applied to remove irrelevant features and to reduce redundancy [22]. In each fold, top 200 features according to the highest relevance‐redundancy indexes were retained. The features that were repeatedly retained in four or five folds were selected for subsequent stepwise forward feature selection. Then, the stepwise forward logistic regression method searched the compact feature subsets by recursively selecting features based on the performance of the models [9]. In this study, the stepwise forward selection (conditional) was used for easy interpretation. The nodal status of tumor was entered as the dependent variable in the corresponding logistic regression model, and the radiomics features selected by the mRMR approach were entered as covariates.

### 2.4 Model Construction and Validation

The selected features were used to construct the logistic regression models. The predicted probabilities of lymph node metastasis were calculated as follows [23]:

$$\begin{array}{c} \mathrm{Pr}=\frac{e\left(\beta_{0}+\beta_{1}X_{1}+\beta_{2}X_{2}+\ldots \right)}{1+e\left(\beta_{0}+\beta_{1}X_{1}+\beta_{2}X_{2}+\ldots \right)}\\ \;=\frac{1}{1+e-\left(\beta_{0}+\beta_{1}X_{1}+\beta_{2}X_{2}+\ldots \right)} \end{array}$$

where Pr is the predicted probabilities of the lymph node metastasis or tumor recurrence. *β*_0_ is the constant. *X*_1_, *X*_2_ … are the independent variables. *β*_1_, *β*_2_ … are the coefficients of corresponding variables.

The predictive accuracy of the binary logistic models was assessed by receiver operating characteristic (ROC) curve analysis. The radiomics signatures were further verified on the independent test dataset. The performance of the radiomics signatures was expressed in terms of accuracy, sensitivity, and specificity. In addition, the highest Youden index was used to optimize the differentiating ability of the models. The predictive accuracy of the multinomial logistic model was assessed by the three‐way confusion matrix. IBM SPSS Statistics software (version 26) was used to perform the abovementioned statistical analysis.

## 3 Results

### 3.1 Patient Characteristics

For the features of lymph node metastasis, the training set (*n* = 219) includes 105 node positive. In the test set (*n* = 24), there are 13 nodes positive. There were no significant differences between the training and test cohorts for nodal status (*p* = 0.0668).

### 3.2 Construction and Validation of the Radiomics Models

#### 3.2.1 For Pretreatment Classification of Nodal Status

**FIGURE 3:**
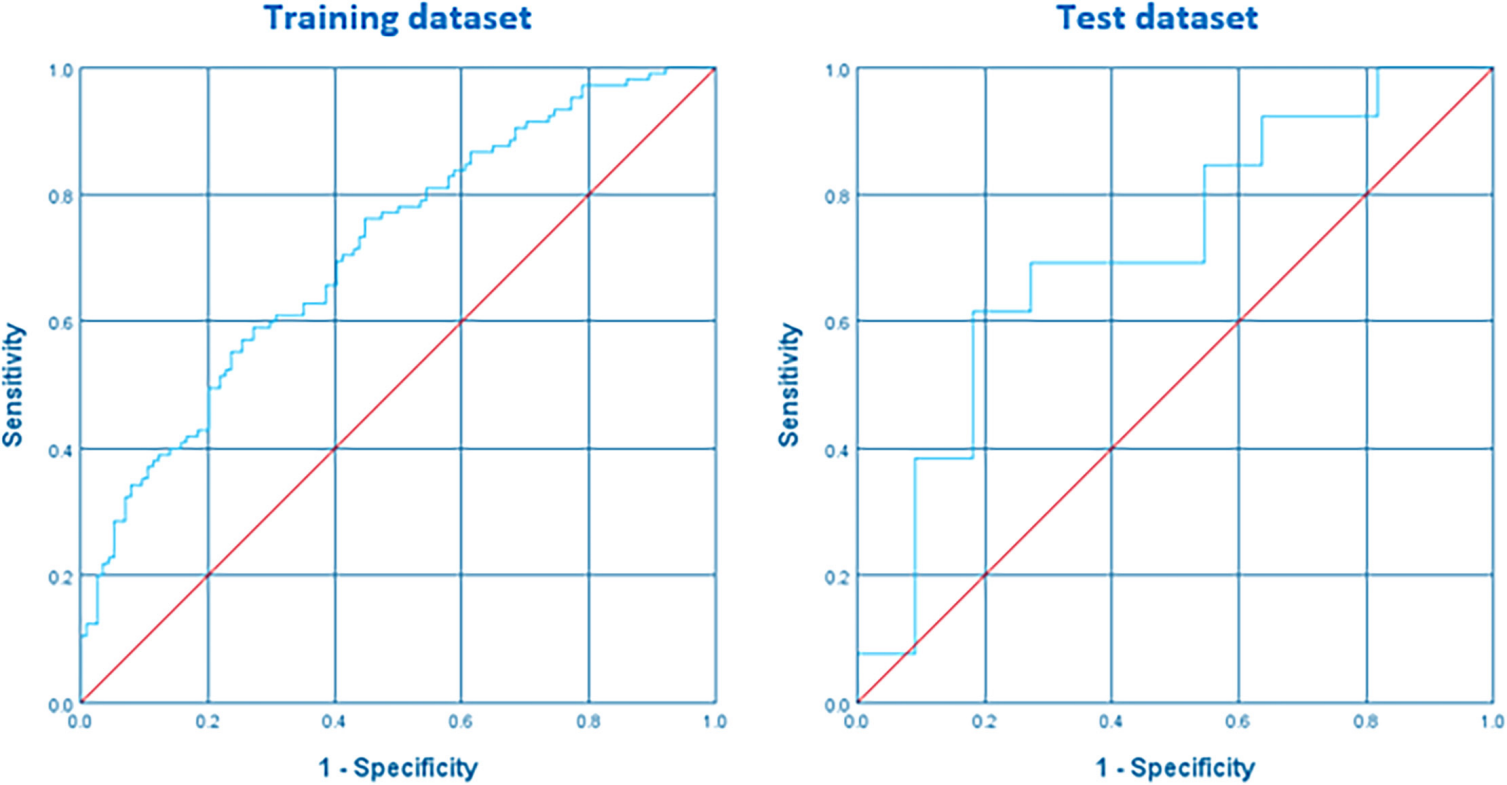
Receiver operating characteristic (ROC) curves of the radiomic model for predicting lymph node metastasis in the training and test sets.

In the training cohort, 474 radiomics features were reduced to 6 potential predictors using the mRMR approach and stepwise forward logistic regression technique. The predicted probability showed a moderate performance for differentiating node‐positive from node‐negative patients, with AUCs (95% CI) of 0.713 (0.646, 0.780) in the training dataset and 0.713 (0.500, 0.926) in the test dataset. The optimal cutoff value of the predicted probability was defined as 0.5 based on the maximum Youden index. The accuracy of the radiomics signature was 66.2% and 66.7% in the training and test groups, respectively (Tables 1 and 2, Figure 3).

**TABLE 1:** List of the selected features for pretreatment classification of nodal status.

Image type	Feature class	Feature name	Coefficient
Wavelet, LL	GLCM	Inverse difference moment normalized (IDMN)	393.360773
Wavelet, HL	NGTDM	Contrast	64.390104
Original	Shape‐based (2D)	Perimeter	0.000914
Wavelet, LH	GLRLM	Run length nonuniformity (RLN)	−0.000129
Wavelet, HL	GLDM	Large dependence emphasis (LDE)	−0.168645
Wavelet, HL	GLDM	Small dependence low gray level emphasis (SDLGLE)	−294.007329
Constant			−384.803085

**TABLE 2:** Diagnostic performance of the radiomic model for pretreatment nodal status classification.

	For classification of nodal status	
	Training data set	Test data set
AUC (95% CI)	0.713 (0.646, 0.780)	0.713 (0.500, 0.926)
Accuracy	66.210%	66.667%
Sensitivity	59.048%	61.539%
Specificity	72.807%	72.727%

Abbreviation: AUC, area under the curve.

#### 3.2.2 Validation of Image Segmentation

The intraobserver and interobserver reproducibility of ROI delineation for 25 randomly selected cases were greater than 0.70 (DSC, 0.701–0.953; Table 3). Therefore, all outcomes were based on the ROIs delineated by the junior sonologist. The interobserver reproducibility of feature extraction was also good (ICC, 0.897–1.000) (Table 4).

**TABLE 3:** Minimum and maximum value of the dice similarity coefficients (DSCs).

	Between first and second delineation by junior sonologist	Between first delineation by junior sonologist and delineation by senior sonologist	Between second delineation by junior sonologist and delineation by senior sonologist
DSC (min, max)	0.801, 0.953	0.701, 0.947	0.770, 0.948

Abbreviation: DSC, dice similarity coefficient.

**TABLE 4:** Intraclass correlation coefficient for radiomics features included in the radiomic signatures.

Radiomic feature	ICC (2, 1) [95% CI]	*P* value
For classification of nodal status (binary model)		
Wavelet‐LL_glcm_Idmn	0.923259 [0.835007, 0.965336]	0.000 (6.3938E‐12)
Wavelet‐HL_ngtdm_Contrast	0.999623 [0.999148, 0.999834]	0.000 (1.7158E‐39)
Original_shape2D_Perimeter	0.896848 [0.637744, 0.962168]	0.000 (3.7753E‐12)
Wavelet‐LH_glrlm_RunLengthNonUniformity	0.988213 [0.973761, 0.994761]	0.000 (2.2768E‐21)
Wavelet‐HL_gldm_LargeDependenceEmphasis	0.994125 [0.980394, 0.997770]	0.000 (1.7369E‐26)
Wavelet‐HL_gldm_SmallDependenceLowGrayLevelEmphasis	0.996929 [0.990633, 0.998796]	0.000 (1.21E‐29)

Abbreviation: ICC, intraclass correlation coefficients.

## 4 Discussion

In the present study, radiomics models were established and validated for preoperative diagnosis of ALN metastasis in BC patients based on CEUS images. This established radiomics CEUS model achieved good prediction performance, which indicates a potential advantage for predicting ALN metastasis in BC (AUC, 0.713 in the test cohort). Several randomized trials [24, 25] suggested that ALN biopsy and SLN biopsy should be omitted in patients without metastatic lymph nodes. Ultrasound‐guided lymph node biopsy has therefore become a standard preoperative assessment in BC patients. However, it has a low sensitivity (50%) in diagnosing SLN involvement because of the inadequate samples [26] and cannot replace SLN biopsy to eliminate its morbidity risk. According to our study's results, the radiomics model was able to detect lymph node metastasis with a higher sensitivity (66.667%) compared with the ultrasound‐guided lymph node biopsy. Although our radiomics signature only achieved a moderate performance (AUC, 0.713 in the test cohort), it is believed that with further optimization, the CEUS‐based radiomics could be developed as an accurate preoperative noninvasive tool for determining nodal status to avoid needless invasive lymph nodes biopsy or excision.

Previously, several mammography, two‐dimensional (2D) ultrasound, CT, and magnetic resonance imaging (MRI) studies have explored the application of radiomics analysis of primary tumors in predicting ALN status. Li, Yang, and Jiao developed a radiomics model based on for preoperative discrimination of dynamic contrast‐enhanced‐MRI for ALN metastasis prediction in BC, and the decision fusion model with clinical information yielded the highest AUC of 0.93 [27]. Wang et al. constructed a radiomics model based on intratumoral and peritumoral radiomics of contrast‐enhanced mammography to predict ALN metastasis in patients with BC, with AUCs of 0.753 and 0.732 in internal and external testing sets, respectively [28]. These studies show that the radiomics model based on mammography, CT, and MRI can achieve good performance in the identification of ALN status in patients with BC. Yu et al.'s study constructed 14 features 2D ultrasound‐based radiomics model to predict ALN metastasis (ALNM) in early‐stage invasive BC, with the AUC of 0.78 and 0.71 in training and validation cohorts [29]. Gao et al.'s report, which have used 2D ultrasound‐based radiomics to predict lymph node malignancy preoperatively, and their models achieved moderate diagnostic accuracy with AUCs of 0.723 in the validation cohort [30]. The previously findings are very similar to our present study. However, they only limited to early stage and invasive cancer. In this study, several types of BC, such as invasive lobular carcinoma, invasive carcinoma of no special type, and ductal carcinoma in situ, as well as advanced stages BC are included. Therefore, the use of our model is more comprehensive. There is also a possibility of combining the use of CEUS‐based and MRI‐based (or CT‐based or mammography‐based) radiomics models in the future development trend.

It is worth noting that the most of the selected features were obtained from wavelet‐decomposed images in this study. The wavelet transform is a mathematical algorithm, which decomposes the original image in both space, and frequency domains [31] could redisplay the tumor characteristics that were previously hidden by speckle in the low‐contrast ultrasound images and showed discriminative ability [32]. Qiu et al., also using wavelet transform to analysis the spatial‐frequency information of the images, showed that the ultrasound‐based radiomics can noninvasively predict ALN metastasis in all stages of breast tumor [33]. Similar results are demonstrated in Qiu et al.'s study. Furthermore, we found that in the binary logistic regression model, an increase in inverse difference moment normalized (IDMN), which indicated a more uniform breast lesion, was associated with increased log‐odds as well as predicted probability of lymph node metastasis. This is likely because lymph node malignancy was more likely to occur in tumors with no internal echo (homogeneous tumors). Previous study demonstrated small dependence low gray level emphasis (SDLGLE) was mentioned as one of the top‐five ranked features for predicting lymph node involvement in an MRI‐based radiomics analysis [34]. Specifically, the feature selection results obtained in our study have demonstrated that SDLGLE is an optimum feature for prediction. ALNM meanwhile, in this study, DSC values of segmentation results between repeated segmentation by junior sonologist, and between two sonologists were greater than 0.700; this probably indicates that the segmentations were very consistent between repeated segmentation and among different sonologists. Therefore, the CEUS‐based radiomics features, which are hard to identify with the naked eye, have the potential to be noninvasive biomarkers for the preoperative prediction of ALNM in BC.

Several limitations can be identified in this study. First, this retrospective study used data collected from single hospital; the sample size in this study was relatively small, which may limit the generalizability of our findings. A larger prospective multi‐institutional study should be conducted for further verification of this preliminary work. Second, the ROIs were manually delineated in this study, which probably yield interobserver variability. Further studies using automatic or semiautomatic segmentation are suggested, although the junior sonologist showed good intraobserver reproducibility and interobserver agreement with the senior sonologist in our study. Finally, BC can be further divided into estrogen receptor‐positive, progesterone receptor‐positive, HER2‐positive, and TN BC based on the receptor status, which are proved to have different prognosis and responses to therapy [35, 36]. However, specific radiomics signature for each subtype was not developed in this study due to the lack of available data and small sample size. Further studies on investigating whether a specific model for each BC subtype would predict BC characteristics more accurately are suggested.

## 5 Conclusion

This project investigated the role of CEUS‐based radiomics in pretreatment BC prediction and proved that the high‐throughput quantitative sonographic features potentially provide additional informative imaging biomarkers and assist clinicians in predicting the ALNM for personalizing therapeutic strategies.

## Author Contributions

**Lawrence Wing Chi Chan and Qiao Hu:** conceptualization, investigation. **Haimei Lun, Mohan Huang, Yihong Zhao, Jingyu Huang, Lingling Li, HoiYing Cheng, Yiki Leung, HongWai So, YuenChun Wong, ChakKwan Cheung, and ChiWang So:** data curation, formal analysis, and investigation. All authors wrote and reviewed the manuscript. All authors have read and agreed to the published version of the manuscript.

## Conflicts of Interest

The authors declare no conflicts of interest.

## Supporting information


**TABLE S1.** Types of ultrasound machines and transducers used in this study.

## Data Availability

All data generated or analyzed during this study are available in this published article and the supporting information files.
