# Supplementary material for: Contrast‐Enhanced Ultrasound‐Based Radiomics for the Prediction of Axillary Lymph Nodes Status in Breast Cancer
Source: Cancer Rep (Hoboken). 2024 Oct 18;7(10):e70011. doi: 10.1002/cnr2.70011 (PMC11488668; doi:10.1002/cnr2.70011)
Supplement: Supplementary file 1 — TABLE S1. Types of ultrasound machines and transducers used in this study. [file CNR2-7-e70011-s001.docx]

TABLE S1: Types of ultrasound machines and transducers used in this study.

| **Types of ultrasound machines used in this study** | **Corresponding transducer** |
| --- | --- |
| LOGIQ E9 scanner (GE Healthcare) | 3-8MHz or 4-15 MHz linear probe |
| Aplio 300 scanner (Canon Medical Systems) | 5-14 MHz linear probe |
| Aplio 500 scanner (Canon Medical Systems) | 5-14 MHz linear probe |
| Aplio i800 scanner (Canon Medical Systems) | 4-18 MHz linear probe |
| RS80A scanner (Samsung Healthcare) | 3-12 MHz linear probe |
| Resona 7 scanner (Midray) | 3-10 MHz or 4-14 MHz linear probe |
| Resona 7OB scanner (Mindray) | 4-14 MHz linear probe |
| Resona 8 scanner (Mindray) | 4-14 MHz linear probe |
| M9 scanner (Mindray) | 3-13 MHz linear probe |
